# Exploring the Analgesic Efficacy and mechanisms of low-dose esketamine in pregnant women undergoing cesarean section: A randomized controlled trial

**DOI:** 10.1016/j.heliyon.2024.e35434

**Published:** 2024-07-30

**Authors:** Junhua Zhang, Dina Sun, Jing Wang, Jie Chen, Yuanjing Chen, Bin Shu, He Huang, Guangyou Duan

**Affiliations:** a Department of Anesthesiology, The Second Affiliated Hospital, Chongqing Medical University, Chongqing, China; b Department of Gynecology and Obstetrics, The Second Affiliated Hospital, Chongqing Medical University, Chongqing, China

**Keywords:** Esketamine, Cesarean section, Hyperalgesia, Inflammation

## Abstract

**Background:**

Postoperative pain is a prevalent concern following a cesarean section. This study aimed to investigate the effect and mechanism of low-dose (0.1 mg/kg) esketamine on postoperative pain management in pregnant women undergoing cesarean sections, specifically in cases where both patient-controlled intravenous analgesia (PCIA) and patient-controlled epidural analgesia (PCEA) were employed.

**Methods:**

Pregnant women intending to undergo elective cesarean section were divided into four subgroups based on the intravenous administration of esketamine and the specific analgesia methods employed: E1 (0.1 mg/kg esketamine + PCEA), E2 (0.1 mg/kg esketamine + PCIA), C1 (saline + PCEA), and C2 (saline + PCIA). The primary outcome was the maximum pain score within 24 h postoperatively. Secondary outcomes included the pressure pain threshold and tolerance at 30 min and 24 h postoperatively, along with the inflammation and adverse event index scores.

**Results:**

A total of 118 pregnant women were assigned to the four groups: E1 (n = 29), E2 (n = 29), C1 (n = 30), and C2 (n = 30). Compared with those in the control groups (C1 + C2), the maximum postoperative pain scores within 24 h in the esketamine groups (E1 + E2) were significantly lower (4 [2–5] vs. 4 [4–6], P = 0.002), and the E1 group exhibited superior analgesic effects compared with other groups. No significant differences were observed in postoperative hyperalgesia or inflammation across the four groups. Notably, esketamine combined with PCIA increased the incidence of postoperative nausea and vomiting (7 [25 %] vs. 0 [0 %]; P = 0.005).

**Conclusion:**

The administration of low-dose (0.1 mg/kg) esketamine effectively alleviates pain following cesarean section, and the analgesic effect is notably enhanced in combination with PCEA. Importantly, these effects do not appear to be mediated through anti-inflammatory mechanisms or the inhibition of hyperalgesia.

**Clinical trial registration number:**

NCT05414006.

## 1 Introduction

Cesarean section (CS) is the most commonly performed surgical procedure [1], and inadequate pain relief following CS is a global concern [2]. Some studies on the severity of acute postoperative pain following CS have reported a high incidence of moderate-to-severe postoperative pain of 39–78 % [3], with severe pain in 20 % of cases [4,5]. Notably, chronic postoperative pain occurs in 18.3 % and 6.8 % of cases at 3 months and 1 year post-CS, respectively [6]. In addition, the mismanagement of perioperative pain is associated with increased opioid use, chronic pain, impaired maternal–fetal bonding, delayed functional recovery, and increased postpartum depression [7]. Patient-controlled epidural analgesia (PCEA) and patient-controlled intravenous analgesia (PCIA) are currently the most commonly used analgesic approaches. High-dose opioids in PCIA exert various adverse effects, such as ileus, constipation, and respiratory depression. Whereas PCEA is limited by complications including motor block and lower extremity venous thrombosis [8]. Consequently, exploring new analgesic approaches and improving the previously established strategies are imperative.

Esketamine, an isomer of ketamine, reduces pain signals induced by nociceptive stimuli in the cortex by inhibiting N-methyl-d-aspartate (NMDA) receptor activation. Several trials have shown that esketamine can relieve pain post-CS [[9], [10], [11], [12]] and attenuate postoperative inflammation [13]. The nociceptive stimuli from the surgical wound are transmitted to the dorsal horn of the spinal cord via the dorsal root ganglion and activate the NMDA receptors to transmit the pain signal to the neural center, resulting in decreased pain tolerance and hyperalgesia [14]. Otherwise, surgical trauma can break down the tissue–organ barrier, causing the release of inflammatory factors and an inflammatory response in the body [15]. Notably, inflammation and hyperalgesia are major causes of postoperative pain. Therefore, based on the mechanisms of inflammation and hyperalgesia and the pharmacology of esketamine, esketamine could be a promising option for postoperative pain following CS.

This randomized controlled trial aimed to explore whether low-dose esketamine can relieve postoperative pain after CS in women who underwent CS with PCIA and PCEA analgesia and compare the effects of the combined application of these two different postoperative analgesia approaches. Additionally, hyperalgesia and inflammatory index evaluations were performed to explore the potential mechanisms.

## 2 Material and methods

### 2.1 Research participants

This prospective, randomized, placebo-controlled, triple-blind clinical trial was approved by the Ethics Committee of the Second Affiliated Hospital of Chongqing Medical University (2022-77). All procedures were performed in accordance with the Declaration of Helsinki guidelines (revised in 2013) and Consolidated Standards of Reporting Trials (CONSORT) reporting guidelines. This trial was registered with clinicaltrials.gov (Identifier: NCT05414006) and conducted at the Second Affiliated Hospital, Chongqing Medical University, a comprehensive teaching hospital. The protocol was explained to all participants, and informed consent was obtained before trial initiation. The clinical data and protocols used in this study are available upon reasonable request from the corresponding author.

### 2.2 Inclusion and exclusion criteria

The trial enrolled patients that met the following criteria: ASA status I–III, age 20–45 years, gestational age 37–42 weeks, and undergoing elective CS with subarachnoid anesthesia. We excluded patients with contraindications for CS, contraindications of combined spinal and epidural anesthesia, severe systemic disease, alcoholism and long-term use of anti-inflammatory and analgesic drugs, contraindications to esketamine or hydromorphone, and those who were unable to cooperate or refused to participate in the trial.

### 2.3 Randomization and masking

In this study, simple randomization with a sealed envelope method was used. A biostatistician who was not responsible for the data management and statistical analysis generated random numbers for the four different groups using SPSS software. Another independent assistant prepared and placed random numbers in opaque, numbered, and sealed envelopes. Pregnant women were enrolled sequentially during the study period and randomly divided into four groups according to the random numbers in the envelopes. A researcher independent of data collection and analysis prepared the interventional drugs and analgesic pumps based on the random numbers. After the intervention, the envelopes were resealed and preserved until the end of the study. The participants, outcome evaluators, and data analysts were blinded throughout the study. The study drugs were prepared preoperatively and assigned based on random computer-generated numbers. Only “study drugs” and the patients' random numbers were marked on the syringes and PCA devices injected after umbilical rupture. The participants, care providers, and outcome assessors were blinded to the allocation. The researchers who analyzed the data were not involved in any treatment or evaluation.

### 2.4 Preoperative and intraoperative management

In this study, all CSs were performed by the same experienced surgical team, with an experience of >1000 CSs. Anesthesia was performed by the same experienced and skilled group of anesthesiologists. After establishing the venous infusion channel of the upper limb, all women received 500 mL of normal saline via an intravenous cannula at a rate of 1 mL/min to keep the vein open. Anesthesiologists monitored the pulse oxygen saturation (SpO_2_), electrocardiogram, and blood pressure. Combined epidural anesthesia was performed at the L2–L3 space with 1.5 mL of 0.75 % bupivacaine injection + cerebrospinal fluid, totaling 2.5 mL. The block level was tested intermittently. If T6 was not reached, 5 mL of 2 % lidocaine was administered epidurally. Close monitoring of maternal vital signs was maintained, and 6 μg of norepinephrine was administered in cases of maternal hypotension (MAP ≤80 % of the MAP base value or an MAP <60 mmHg). In cases of bradycardia (ventricular rate <50 BPM), 0.3 mg of atropine was administered, and in cases of maternal SpO_2_ < 90 % or respiratory distress, 60 % oxygen was given via a mask. A Pfannenstiel incision with peritoneal closure was performed in all CSs. Immediately after umbilical rupture, 20 UI of oxytocin was injected (500 mL, 5 % glucose solution) at a rate of 100 mL/h. Dexamethasone (10 mg), metoclopramide (5 mg), and ondansetron (8 mg) were routinely administered to prevent postoperative nausea and vomiting.

### 2.5 Intervention and allocation

All participants were randomly divided into four groups at a ratio of 1:1:1:1 as follows: the E1 group received esketamine at a dose of 0.1 mg/kg intravenously after delivery, followed by PCEA (200 mL containing 10 μg/mL of hydromorphone + 0.11 % ropivacaine). The E2 group received esketamine at a dose of 0.1 mg/kg intravenously after delivery, followed by postoperative PCIA (100 mL containing 100 μg/mL of hydromorphone). The C1 group received a placebo intravenously after delivery, followed by PCEA (200 mL containing 10 μg/mL hydromorphone + 0.11 % ropivacaine). The C2 group received a placebo intravenously after delivery, followed by PCIA (100 mL containing 100 μg/mL hydromorphone).

For PCEA, patients received an initial dose of 6 mL, delivered in pulses of 6 mL/h thereafter, with intermittent boluses of 6 mL. Lockout time was set at 30 min, with a limit of 18 mL/h. For PCIA, patients received an initial dose of 20 μg/kg, delivered slowly over 10 min. The PCIA pump was programmed for continuous background infusion at a rate of 3 μg/kg/h, with an additional 3 μg/kg provided with each button press. A lockout interval was set at 10 min, with a maximum limit of 20 μg/kg/h. In instances where analgesic consumption reached the maximum hourly dose and the Numerical Rating Scale (NRS) score was ≥4, intravenous tramadol at a dose of 50 mg was administered for rescue analgesia.

### 2.6 Outcome measurement

The demographic characteristics (age, height, weight, and gestational age) and comorbidities of the patients were recorded. The primary outcome of this study was the maximum pain NRS score within 24 h postoperatively, expressed as the NRS score (0 = no pain and 10 = most painful), evaluated by a researcher blinded to the group allocation. Maximum rest incision pain, moving incision pain, and visceral pain scores were evaluated at 0–6 h, 6–12 h, and 12–24 h postoperatively. Further, the area under the curve (AUC) of the NRS pain score at different time points was also calculated.

Upon admission, all patients underwent assessments using the Generalized Anxiety Disorder-7 (GAD-7) scale and the Edinburgh Postnatal Depression Scale (EPDS) to evaluate the presence of anxiety and depression. Preoperative measurements included the neutrophil count (N), neutrophil-to-lymphocyte ratio (NLR), and white blood cell count (WBC). After entering the operating room, pressure pain threshold and tolerance were measured using an algometer placed on the forearm of the dominant hand. Each measurement was separated by a period of at least 3 min, and the average of these three measurements was considered the baseline value.

Secondary outcome measures of this study included the pressure pain threshold and tolerance at 30 min and 24 h postoperatively; number of patient-controlled analgesia pumps pressed; patient-controlled analgesia pump consumption; side effects of drugs, including dizziness, postoperative nausea and vomiting (PONV), drowsiness, irritability, itching, hallucinations, nightmares, diplopia, hypertension, and tachycardia; serious adverse events such as respiratory failure requiring oxygen therapy or ventilator support; severe hemodynamic disorders (heart rate <45 beats per min, systolic blood pressure <80 mmHg, or cardiac arrest); and severe neurological impairment (convulsions and coma).

In this study, the hyperalgesia assessments were conducted using an FDIX 25 algometer (Zhiji, DS2-100N, Zhiji Precision Instruments Co., Ltd., Dongguan, China [in kgf]). The procedure involved vertical contact with the skin, with pressure gradually increasing until the patient reported pain, at which point the pressure pain threshold was recorded. Pressure was further increased until the patient could no longer tolerate it, and the pressure pain tolerance was recorded. The average value was measured on the skin on the inner forearm at the front crease of the elbow at 3, 6, and 9 cm. The preoperative (T0) value was used as the baseline, and the values were measured at 30 min (T1) and 24 h (T2) postoperatively. The extent of change relative to the T0 value indicated the degree of hyperalgesia.

### 2.7 Statistical analysis

The maximum postoperative pain NRS score within 24 h based on the pre-experimental study was 5.2 ± 2.0 when esketamine was not administered, and the NRS score of esketamine prophylactics was 3.9 ± 1.8. The required sample size of 46 cases for each group was determined using the PASS sample size calculation software at a significance level of 0.05 and a power of 90 % according to the 1:1 parallel control difference test design. The final sample size was calculated as 58 patients for each group, considering a 20 % attrition rate. Consequently, this study included 60 patients in each group, with 30 patients in each subgroup, accounting for 120 patients in total.

Data analysis was performed using IBM SPSS Statistics version 22.0 (IBM Corp., Armonk, N.Y., USA). A general descriptive method was adopted based on the type of data. Continuous variables with a normal distribution were presented as the mean ± standard deviation, whereas quantitative data with a non-normal distribution were presented as the median (interquartile interval), and the quantitative percentage was used for qualitative variables. According to the data type, baseline data between patients in groups C and E were compared using an independent sample *t*-test, Mann–Whitney *U* test, or Chi-square test. Maximum pain NRS, rest incision pain, moving incision pain, and visceral pain score AUCs within 24 h postoperatively were compared using an independent sample *t*-test or Mann–Whitney *U* test. Adverse events between groups C and E were compared using a chi-square test. Exploratory subgroup analysis between groups E1 and C1 and E2 and C2 was also performed using an independent sample *t*-test or Mann–Whitney *U* test. Additionally, a multiple linear regression analysis was performed to investigate the influence of esketamine use (yes or no) and the choice of analgesic method (PCIA or PCEA) on postoperative pain intensity, including maximum pain NRS, rest incision pain, moving incision pain, and visceral pain score AUCs within 24 h postoperatively. A two-sided P < 0.05 was considered statistically significant.

## 3 Results

**Fig. 1:**
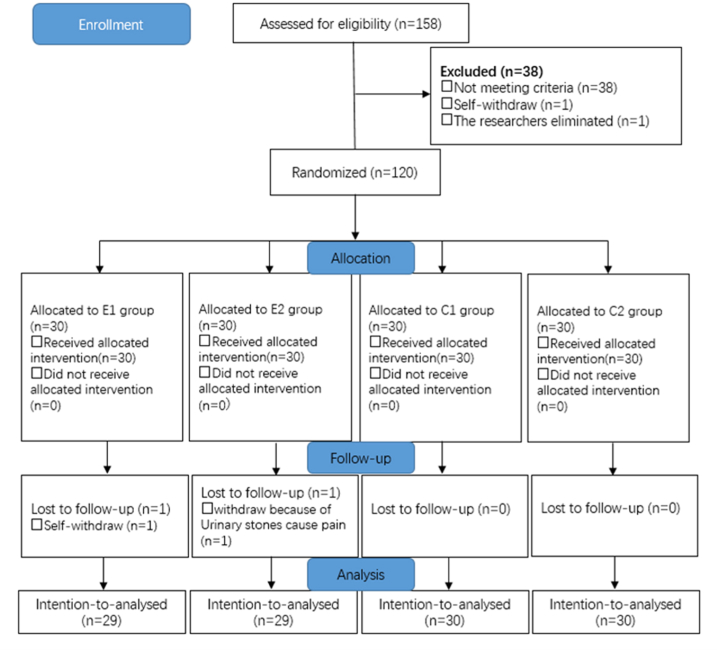
CONSORT flow chart of the study.

In this study, 158 pregnant women were screened for eligibility. Of these, 120 women who met the inclusion criteria were successfully enrolled. The final visit for the last randomized participant was concluded on August 2, 2022. Throughout the study duration, the blinding process remained uninterrupted. Two women were ultimately excluded from the trial, with one withdrawing voluntarily and another being excluded due to pain resulting from urinary stones. Consequently, 118 pregnant women were randomly assigned to groups E1 (n = 29), E2 (n = 29), C1 (n = 30), or C2 (n = 30) (Fig. 1), and all were included in the final per-group analyses (Fig. 1). No significant differences were observed in the demographic characteristics of the four groups (Table 1).

**Table 1:** Baseline demographic characteristics of patients in different groups.

	E1 (n = 29)	E2 (n = 29)	C1 (n = 30)	C2 (n = 30)
**Age (years)**	31.2 ± 3.4	31.4 ± 4.0	32 ± 4.1	30.3 ± 4.3
**Height (cm)**	159.6 ± 4.3	157.5 ± 5.5	158.9 ± 3.9	160.9 ± 6.1
**Weight (kg)**	68.9 ± 10.2	68.4 ± 10.3	67.9 ± 7.9	69.1 ± 8.9
**Body max index (kg/m**^**2**^**)**	27.0 ± 3.3	27.5 ± 3.7	26.9 ± 3.0	26.7 ± 3.2
**Gestation age (week)**	38.2 ± 1.1	37.7 ± 0.8	38.3 ± 0.9	38.5 ± 0.6
**Surgical duration (min)**	46.1 ± 18.2	44.6 ± 14.4	45.7 ± 14.7	45.2 ± 11.5
**Preoperative WBC ( × 10**^**9**^**/L)**	8.2 ± 2.1	8.3 ± 2.1	8.2 ± 1.9	8.0 ± 1.6
**Preoperative N ( × 10**^**9**^**/L)**	6.2 ± 1.8	6.1 ± 1.8	6.1 ± 1.6	6.0 ± 1.4
**Preoperative NLR**	4.4 ± 1.7	4.0 ± 1.2	4.3 ± 1.2	4.3 ± 1.3
**PPT-T0 (kg)**	0.7 ± 0.3	0.7 ± 0.2	0.7 ± 0.2	0.7 ± 0.3
**PTO-T0 (kg)**	1.8 ± 0.5	1.7 ± 0.5	2.0 ± 0.7	1.8 ± 0.8
**Gestational diabetes mellitus, n (%)**	6 (21.4 %)	9 (32.1 %)	7 (25.9 %)	6 (21.4 %)
**Hypothyroidism, n (%)**	4 (14.3 %)	3(10.7 %)	1 (3.7 %)	6 (21.4 %)
**Hypertension, n (%)**	1 (3.6 %)	2 (7.1 %)	2 (7.4 %)	2 (7.1 %)
**Hyperproteinemia, n (%)**	2 (7.1 %)	3 (10.7 %)	0 (0 %)	3 (10.7 %)
**Prior history of CS, n (%)**	12 (42.9 %)	10 (35.7 %)	13 (48.1 %)	7 (25 %)
**Depression, n (%)**	4 (13.8 %)	2 (6.9 %)	3 (10 %)	2 (6.7 %)
**Anxiety, n (%)**	4 (13.8 %)	5 (17.2 %)	7 (23.3 %)	5 (16.7 %)

Data are presented as the mean (SD) or number (proportion).

Abbreviations: WBC, white blood cell; N, neutrophil; NLR, neutrophil-to-lymphocyte ratio; PPT-T0, preoperative pressure pain threshold; PTO-T0, preoperative pressure pain tolerance.

Compared with patients in group C (C1 and C2), those in group E (E1 and E2) exhibited a statistically significant reduction in the maximum postoperative pain score within 24 h, the AUCs for rest incision pain, moving incision pain, and visceral pain (Table 2). The maximum postoperative pain score within 24 h and the AUCs for rest incision pain and moving incision pain were higher in group C1 than in group E1. Furthermore, the maximum postoperative pain score within 24 h and the AUC for visceral pain were higher in group E2 compared with group C2 (Table 2).

**Table 2:** Comparisons of primary outcomes related to pain between different groups.

	Maximum pain score 24 h postoperatively	Rest incision pain-AUC	Visceral pain-AUC	Moving incision pain-AUC
Group E (n = 58)	3.7 ± 1.5	9 (0–19.5)	39 (12–66)	66 (36–91.5)
Group C (n = 60)	4.6 ± 1.5	21 (6–36)	66 (36–90)	84 (60–102)
P-value	0.002	0.006	0.005	0.010
Group E1 (n = 29)	3(2–4)	6 (0–12)	18 (0–42)	36 (24–66)
Group C1 (n = 30)	4 (3–4.3)	12 (0–24)	39 (13.5–61.5)	66 (54–90)
P-value	0.012	0.013	0.067	0.002
Group E2 (n = 29)	4.5 ± 1.2	18 (6–39)	66 (30–87)	90 (66–102)
Group C2 (n = 30)	5.3 ± 1.1	33 (10.5–54)	84 (66–108)	90 (82.5–114)
P-value	0.008	0.108	0.010	0.386

Data are presented as the mean (±) standard deviation and interquartile range.

Abbreviation: AUC: Area under curve.

**Fig. 2:**
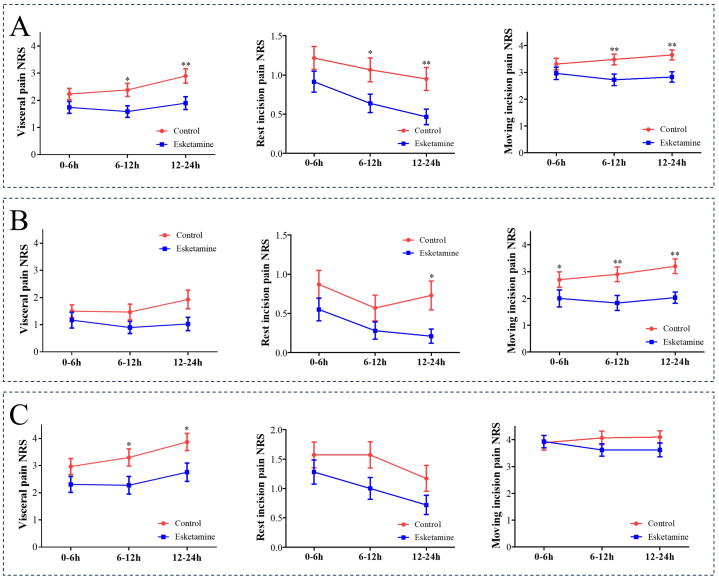
Comparisons of postoperative pain NRS between esketamine and control groups. A: Comparison between the esketamine groups (E1 and E2) and the control groups (C1 and C2). B: Comparison between groups E1 and C1 in patients administered PCEA. C: Comparison between groups E2 and C2 in patients administered PCIA. (NRS: number rating scale).

The NRS scores of rest incision, visceral, and moving incision pain were significantly lower in group E (E1 and E2) than in group C (C1 and C2) at 6–12 h and 12–24 h postoperatively (Fig. 2A). In the intergroup analysis of PCEA, moving incision pain at 6–12 h and rest incision and moving pain at 12–24 h postoperatively were higher in group C (Fig. 2B). In the intergroup analysis of PCIA, 6–12 h and 12–24 h postoperative visceral pain were higher in group C than in group E (Fig. 2C).

Various factors were examined in the intergroup analyses of PCEA (Table 3) and PCIA (Table 4). Notably, no significant difference was observed in the hyperalgesia index, including preoperative pressure pain threshold, preoperative pressure pain tolerance, inflammation index, including N, NLR, and WBC count, and in terms of adverse reactions such as dizziness and drowsiness (Table 5).

**Table 3:** Comparisons of other outcomes between groups E1 and C1 using PCEA.

	Group E1 (n = 29)	Group C1 (n = 30)	P-value
PPT -T1 (kgf)	0.6 ± 0.3	0.6 ± 0.2	0.644
PPT -T2 (kgf)	0.7 ± 0.3	0.7 ± 0.2	0.869
PTO -T1 (kgf)	1.3 ± 0.6	1.4 ± 0.6	0.475
PTO -T2 (kgf)	1.6 ± 0.4	1.7 ± 0.7	0.316
PPT ratio-T1	1.0 ± 0.4	0.9 ± 0.2	0.327
PPT ratio -T2	1.1 ± 0.4	1.1 ± 0.3	0.502
PTO ratio-T1	0.7 ± 0.2	0.7 ± 0.2	0.895
PTO ratio-T2	0.9 ± 0.2	0.9 ± 0.3	0.863
Analgesic consumption (mL)	148.2 ± 14.0	152.2 ± 15.0	0.310
Time to feel pain (h)	4 (2–8)	4 (1.5–5.5)	0.782
Number of compressions of PCEA (times)	1 (0–1.75)	0 (0–2)	0.652
Postoperative WBC ( × 10^9^/L)	10.9 ± 2.6	10.7 ± 3.1	0.818
Postoperative N ( × 10^9^/L)	8.8 ± 2.4	8.6 ± 3.0	0.789
Postoperative NLR	7.4 ± 3.6	6.4 ± 2.4	0.228
Incidence of severe contraction pain n (%)	1 (3.6 %)	1 (3.7 %)	0.979
Incidence of severe incision pain n (%)	1 (3.6 %)	3 (11.1 %)	0.282
Incidence of severe pain n (%)	1 (3.6 %)	4 (14.8 %)	0.147

Data are presented as the mean (SD), number (proportion), or mean (interquartile range).

PPT ratio-T1 = PPT-T1/PPT-T0; PPT ratio-T2 = PPT-T2/PPT-T0; PTO ratio-T1 = PTO-T1/PTO-T0; PTO ratio-T2 = PTO-T2/PTO-T0.

Abbreviations: WBC, white blood cell; N, neutrophil; NLR, neutrophil-to-lymphocyte ratio; PPT-T0, preoperative pressure pain threshold; PTO-T0, preoperative pressure pain tolerance.

**Table 4:** Comparisons of other outcomes between groups E2 and C2 using PCIA.

	Group E2 (n = 29)	Group C2 (n = 30)	P-value
PPT -T1 (kgf)	0.6 ± 0.2	0.6 ± 0.1	0.383
PPT -T2 (kgf)	0.8 ± 0.2	0.7 ± 0.3	0.307
PTO -T1 (kgf)	1.5 ± 0.4	1.3 ± 0.5	0.288
PTO -T2 (kgf)	1.6 ± 0.4	1.6 ± 0.6	0.759
PPT Ratio-T1	0.9 ± 0.3	0,9 ± 0.3	0.778
PPT Ratio -T2	1.2 ± 0.4	1.2 ± 0.4	0.711
PTO Ratio-T1	0.9 ± 0.2	0.8 ± 0.2	0.134
PTO Ratio-T2	1.0 ± 0.3	1.0 ± 0.3	0.739
Analgesic consumption (mL)	57.4 ± 9.5	59.8 ± 10.0	0.35
Time to feel pain (h)	1.625 (1–3.25)	1.625 (0.95–2.5)	0.555
Number of compressions of PCIA (times)	1 (0–3.5)	2 (0–3)	0.278
Postoperative WBC ( × 10^9^/L)	11.0 ± 3.4	10.5 ± 1.6	0.464
Postoperative N ( × 10^9^/L)	8.8 ± 2.9	8.3 ± 1.6	0.360
Postoperative NLR	7.1 ± 2.9	6.2 ± 2.3	0.195
Incidence of severe contraction pain, n (%)	3 (10.7 %)	7 (25 %)	0.163
Incidence of severe incision pain, n (%)	5 (17.9 %)	8 (28.6 %)	0.342
Incidence of severe pain, n (%)	6 (21.4 %)	11 (39.3 %)	0.146

Data are presented as the mean (SD), number (proportion), or mean (interquartile range).

PPT ratio-T1 = PPT-T1/PPT-T0; PPT ratio-T2 = PPT-T2/PPT-T0; PTO ratio-T1 = PTO-T1/PTO-T0; PTO ratio-T2 = PTO-T2/PTO-T0.

Abbreviations: WBC, white blood cell; N, neutrophil; NLR, neutrophil-to-lymphocyte ratio; PPT-T0, preoperative pressure pain threshold; PTO-T0, preoperative pressure pain tolerance.

**Table 5:** Comparisons of adverse reactions between groups E and C.

	Group E	Group C	P-value
Dizzy, n (%)	11 (22.9 %)	9 (18 %)	0.546
Drowsiness, n (%)	23 (47.9 %)	19(38 %)	0.321
Nausea and vomiting, n (%)	11 (22.9 %)	6 (12 %)	0.154
Hallucinations, n (%)	1 (2.1 %)	0(0 %)	0.490
Nightmare, n (%)	0 (0 %)	0(0 %)	NA

Data are presented as the number (proportion).

**Fig. 3:**
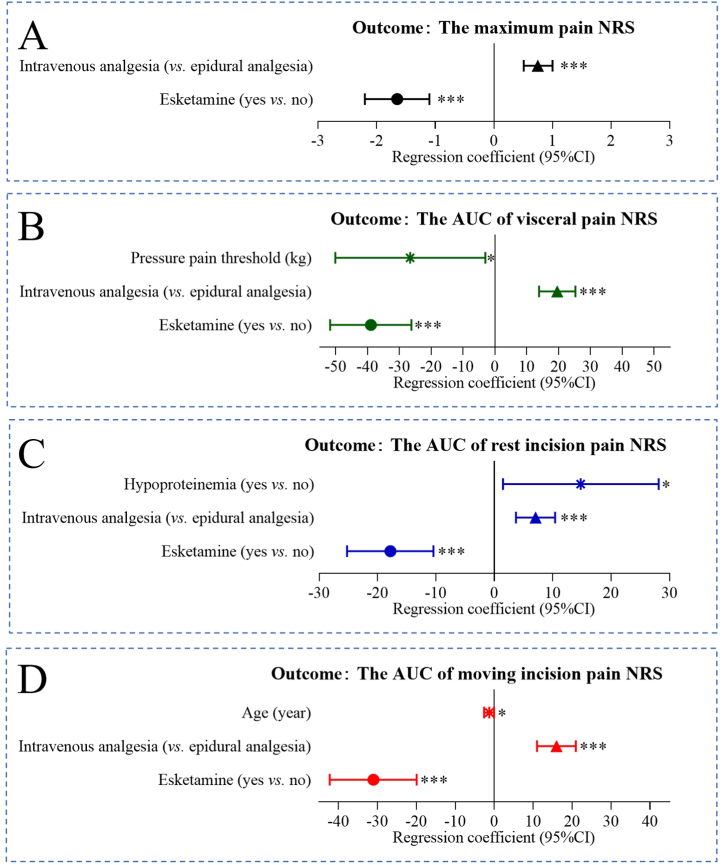
Regression analysis of influencing factors of maximum pain NRS (A) AUC of visceral pain NRS (B), AUC of rest incision pain NRS (C), and moving incision pain NRS (D). (AUC, area under the curve; NRS, numerical rating scale; CI, confidence interval).

The multiple linear regression analysis revealed that the factors affecting the maximum postoperative pain score within 24 h were PCIA (0.754 [95%CI: 0.51 to 0.999]) and esketamine (˗1.648 [95%CI ˗2.195 to ˗1.102]) (Fig. 3A). Preoperative pain threshold (˗26.553 [95%CI ˗50.135 to ˗2.971]), esketamine (˗38.970 [95%CI ˗51.711 to ˗26.230]), and PCIA (19.634 [95%CI 13.940 to 25.238]) affected the postoperative visceral pain NRS score (Fig. 3B). In NRS analysis for rest incision pain, the influencing factors were esketamine (˗17.814 [95%CI ˗25.215 to ˗10.413]), preoperative hypoproteinemia (14.816 [95%CI 1.495 to 28.137]), and PCIA (7.074 [95%CI 3734 to 10.414]) (Fig. 3C). In the moving incision pain NRS analysis, esketamine (˗31.030 [95%CI ˗42.158 to ˗19.902]), maternal age (˗1.301 [95%CI ˗2.559 to ˗0.44]) and PCIA (15.983 [95%CI 11.002 to 20.963]) were influencing factors (Fig. 3D).

## 4 Discussion

The primary outcomes of this investigation offer several key insights. First, a low dose of esketamine (0.1 mg/kg) plays a significant role in alleviating pain post-CS. Second, esketamine in combination with PCEA is a more effective approach for managing acute postoperative pain. Third, the mechanism underlying the analgesic effect of this low esketamine dosage does not appear to be rooted in the inhibition of hyperalgesia or its anti-inflammatory properties. Lastly, esketamine in combination with PCIA does lead to an increased incidence of PONV. PCEA and PCIA with opioids are common analgesic methods used after a CS. High-dose opioids cause several reactions, such as a high pain tolerance and hyperalgesia, weakening the analgesic effect. PCEA reduces pain and stress reactions caused by trauma and may promote early recovery. Therefore, PCEA is significantly superior to PCIA, and this hypothesis has also been tested in other abdominal surgeries [16,17]. Esketamine can exert complementary analgesic effects, emerging as a superior choice in combination with PCEA for acute pain management. An increasing body of evidence suggests that perioperative esketamine administration during CS can effectively relieve postoperative pain [9,10,[18], [19], [20], [21], [22], [23], [24]]. In most previous studies, the dose of esketamine was ≥0.2 mg/kg, with some inevitable adverse reactions [19,25]. Therefore, in this study, we selected a single low-dose intravenous injection of 0.1 mg/kg to explore its effects in relieving pain post-CS. The exact mechanism through which low-dose esketamine alleviates post-CS pain remains uncertain. Surgical trauma induces a robust inflammatory response, causing inflammatory pain and noxious stimulation that activates NMDA receptors through the dorsal root ganglion, amplifying pain signals reaching the cerebral cortex and leading to hyperalgesia. Consequently, we hypothesized that esketamine may attenuate pain signal transduction and inhibit hyperalgesia by noncompetitively antagonizing NMDA receptors, thereby reducing postoperative pain. A prior study demonstrated the ability of esketamine to inhibit hyperalgesia following thyroidectomy. However, in thyroidectomy, hyperalgesia results from both surgical trauma and perioperative opioid use.

In our study, we used an algometer to detect changes in the PPT and PTO levels at three time points to reflect the occurrence and development of hyperalgesia. Notably, a decrease in PPT and PTO occurred in all four groups at 30 min postoperatively, indicating the occurrence of hyperalgesia. Nevertheless, no statistical difference was found in the intergroup analysis, which may be explained as follows: 1) The analgesic effect of low-dose esketamine may not be attributed to the inhibition of hyperalgesia, and 2) the inhibitory effect of esketamine on trauma-induced hyperalgesia might not be as pronounced as that of opioids.

Ketamine and esketamine reduce perioperative inflammation [13,[26], [27], [28], [29], [30]], and the NLR ratio may reflect systemic inflammation and stress [[31], [32], [33]]. In our analysis, we compared NLR, N, and WBC levels among the various subgroups both preoperatively and at 2 days postoperatively. However, no significant differences among the subgroups indicated that the potential mechanism of the analgesic effect of low-dose esketamine was not anti-inflammatory.

In addition, our findings revealed that PCEA is significantly better than PCIA, which is consistent with the results of previous studies [[34], [35], [36], [37], [38], [39]]. Gauger et al., in a randomized controlled trial on the analgesic effects of hydromorphine combined with PCEA and PCIA after posterior spinal cord fusion, reported that PCEA was significantly better than PCIA [40]. In our study, the PCEA drugs were similar to those used by Gauger et al., further validating the superior effects of PCEA to PCIA for analgesia after cesarean delivery.

This study had some limitations. First, we did not conduct a follow-up assessment of depression and anxiety among the women in puerperia in the postoperative period, which should be explored in the future. Second, we did not explore different doses of esketamine to determine the optimal dose. Third, we did not record additional analgesia, and the effects of esketamine on the incidence of additional analgesia should be further explored in future studies. Finally, this study only evaluated acute pain in pregnant women within 24 h postoperatively, and the effect of esketamine on the development of chronic pain after CS remains unexplored.

## 5 Conclusions

Intraoperatively administered 0.1 mg/kg esketamine could effectively relieve pain after CS, with the superior analgesic effect of esketamine combined with PCEA. However, the underlying mechanism is not related to the inhibition of hyperalgesia and anti-inflammation and needs to be confirmed in further studies.

### 5.1 Ethics statement

This clinical trial was approved by the Ethics Committee of the Second Affiliated Hospital of Chongqing Medical University (2022-77). All procedures were performed in accordance with the Declaration of Helsinki guidelines (revised in 2013) and Consolidated Standards of Reporting Trials (CONSORT) reporting guidelines.

### 5.2 Clinical trial

The clinical trial described in this paper was registered at clinicaltrials.gov under the registration number NCT05414006.

## Data availability statement

The original contributions presented in this study are included in the article/supplementary material, and further inquiries can be directed to the corresponding authors.

## Source of funding

This work was supported by the Senior Medical Talents Program of Chongqing for Young and Middle-aged.

## CRediT authorship contribution statement

**Junhua Zhang:** Writing – original draft. **Dina Sun:** Investigation. **Jing Wang:** Methodology. **Jie Chen:** Resources. **Yuanjing Chen:** Data curation. **Bin Shu:** Writing – review & editing. **He Huang:** Project administration. **Guangyou Duan:** Writing – review & editing.

## Declaration of competing interest

The authors declare that they have no known competing financial interests or personal relationships that could have appeared to influence the work reported in this paper.

## References

[1] Molina G. (2015). Relationship between cesarean delivery rate and maternal and neonatal mortality. JAMA.

[2] Carvalho B. (2005). Patient preferences for anesthesia outcomes associated with cesarean delivery. Anesth. Analg..

[3] Karlstrom A. (2007). Postoperative pain after cesarean birth affects breastfeeding and infant care. J. Obstet. Gynecol. Neonatal Nurs..

[4] Guevara J. (2021). Predicting pain after Cesarean delivery: pressure algometry, temporal summation, three-item questionnaire. Can. J. Anaesth..

[5] Gamez B.H., Habib A.S. (2018). Predicting severity of acute pain after cesarean delivery: a narrative review. Anesth. Analg..

[6] Jin J. (2016). Prevalence and risk factors for chronic pain following cesarean section: a prospective study. BMC Anesthesiol..

[7] Eisenach J.C. (2008). Severity of acute pain after childbirth, but not type of delivery, predicts persistent pain and postpartum depression. Pain.

[8] Carvalho B., Butwick A.J. (2017). Postcesarean delivery analgesia. Best Pract. Res. Clin. Anaesthesiol..

[9] Wang Y. (2022). Effect of low-dose esketamine on pain control and postpartum depression after cesarean section: a retrospective cohort study. Ann. Palliat. Med..

[10] Shen J. (2022). The effect of low-dose esketamine on pain and post-partum depression after cesarean section: a prospective, randomized, double-blind clinical trial. Front Psychiatry.

[11] Bornemann-Cimenti H., Wejbora M., Michaeli K., Edler A., Sandner-Kiesling A. (2016). The effects of minimal-dose versus low-dose S-ketamine on opioid consumption, hyperalgesia, and postoperative delirium: a triple-blinded, randomized, active- and placebo-controlled clinical trial. Minerva Anestesiol..

[12] Lei Y. (2021). Effects of esketamine on acute and chronic pain after thoracoscopy pulmonary surgery under general anesthesia: a multicenter-prospective, randomized, double-blind, and controlled trial. Front. Med..

[13] Zhang J. (2023). S-Ketamine attenuates inflammatory effect and modulates the immune response in patients undergoing modified radical mastectomy: a prospective randomized controlled trial. Front. Pharmacol..

[14] Zhang Y. (2021). Structural basis of ketamine action on human NMDA receptors. Nature.

[15] Margraf A. (2020). Systemic inflammatory response syndrome after surgery: mechanisms and protection. Anesth. Analg..

[16] Falk W. (2021). Comparison between epidural and intravenous analgesia effects on disease-free survival after colorectal cancer surgery: a randomised multicentre controlled trial. Br. J. Anaesth..

[17] Li J. (2019). Efficacy and safety of patient-controlled analgesia compared with epidural analgesia after open hepatic resection. Ann. Surg..

[18] Wang W. (2022). Effects of esketamine on analgesia and postpartum depression after cesarean section: a randomized, double-blinded controlled trial. Medicine (Baltim.).

[19] Xu L.L. (2023). Efficacy and safety of esketamine for supplemental analgesia during elective cesarean delivery: a randomized clinical trial. JAMA Netw. Open.

[20] Suppa E. (2012). A study of low-dose S-ketamine infusion as "preventive" pain treatment for cesarean section with spinal anesthesia: benefits and side effects. Minerva Anestesiol..

[21] Li S. (2024). Efficacy of esketamine for the treatment of postpartum depression and pain control following cesarean section: a randomized, double-blind, controlled clinical trial. BMC Anesthesiol..

[22] Lou F. (2023). Analysis of the analgesic effect, emotion, and safety of esketamine in cesarean section analgesia for puerperae. Altern Ther Health Med.

[23] Jiang M., Xu J. (2024). A study on the effects of esketamine combined with comprehensive nursing intervention on postoperative pain, postpartum depression, and quality of life in women undergoing cesarean section. Altern. Ther. Health Med..

[24] Guo Y. (2023). Analgesic effect of esketamine combined with tramadol for patient-controlled intravenous analgesia after cesarean section: a randomized controlled trial. J. Pain Res..

[25] Chen Y. (2023). Safety and tolerability of esketamine in propofol based sedation for endoscopic variceal ligation with or without injection sclerotherapy: randomized controlled trial. Dig. Endosc..

[26] Halaris A., Cook J. (2023). The glutamatergic system in treatment-resistant depression and comparative effectiveness of ketamine and esketamine: role of inflammation?. Adv. Exp. Med. Biol..

[27] Johnston J.N. (2023). Inflammation, stress and depression: an exploration of ketamine's therapeutic profile. Drug Discov. Today.

[28] Nowak W. (2019). Pro-inflammatory monocyte profile in patients with major depressive disorder and suicide behaviour and how ketamine induces anti-inflammatory M2 macrophages by NMDAR and mTOR. EBioMedicine.

[29] Wang T. (2022). Esketamine alleviates postoperative depression-like behavior through anti-inflammatory actions in mouse prefrontal cortex. J. Affect. Disord..

[30] Welters I.D. (2011). Continuous S-(+)-ketamine administration during elective coronary artery bypass graft surgery attenuates pro-inflammatory cytokine response during and after cardiopulmonary bypass. Br. J. Anaesth..

[31] Zahorec R. (2021). Neutrophil-to-lymphocyte ratio, past, present and future perspectives. Bratisl. Lek. Listy.

[32] Serra R. (2021). Neutrophil-to-lymphocyte ratio and platelet-to-lymphocyte ratio as biomarkers for cardiovascular surgery procedures: a literature review. Rev. Recent Clin. Trials.

[33] Perry L.A. (2022). Perioperative neutrophil-lymphocyte ratio predicts mortality after cardiac surgery: systematic review and meta-analysis. J. Cardiothorac. Vasc. Anesth..

[34] Halpern S.H. (2004). A multicenter randomized controlled trial comparing patient-controlled epidural with intravenous analgesia for pain relief in labor. Anesth. Analg..

[35] Hwang B.Y. (2018). Comparison of patient-controlled epidural analgesia with patient-controlled intravenous analgesia for laparoscopic radical prostatectomy. Korean J Pain.

[36] Lu S. (2015). Comparison of pain relief between patient-controlled epidural analgesia and patient-controlled intravenous analgesia for patients undergoing spinal fusion surgeries. Arch Orthop Trauma Surg.

[37] Li Y. (2016). Effects of patient-controlled epidural analgesia and patient-controlled intravenous analgesia on analgesia in patients undergoing spinal fusion surgery. Am J Ther.

[38] Ferguson S.E. (2009). A prospective randomized trial comparing patient-controlled epidural analgesia to patient-controlled intravenous analgesia on postoperative pain control and recovery after major open gynecologic cancer surgery. Gynecol. Oncol..

[39] Zhang X.P. (2024). Efficacy and safety of patient-controlled epidural analgesia versus patient-controlled intravenous analgesia following open hepatectomy: a single-center retrospective study. Heliyon.

[40] Gauger V.T. (2009). Epidural analgesia compared with intravenous analgesia after pediatric posterior spinal fusion. J. Pediatr. Orthop..

